# An exploratory study on risk factors for chronic non-communicable diseases among adolescents in Malaysia: overview of the Malaysian Health and Adolescents Longitudinal Research Team study (The MyHeART study)

**DOI:** 10.1186/1471-2458-14-S3-S6

**Published:** 2014-11-24

**Authors:** Majid Abdul Hazreen, Tin Tin Su, Muhammad Yazid Jalaludin, Maznah Dahlui, Karuthan Chinna, Maslinor Ismail, Liam Murray, Marie Cantwell, Nabilla Al Sadat, MyHeART Study Group

**Affiliations:** 1 Centre for Population Health (CePH) and Department of Social & Preventive Medicine, Faculty of Medicine, University Malaya, 50603 Kuala Lumpur, Malaysia; 2 Department of Paediatrics, Faculty of Medicine, University Malaya, 50603 Kuala Lumpur, Malaysia; 3 Julius Centre University of Malaya (JCUM), Department of Social and Preventive Medicine Faculty of Medicine, University Malaya, 50603 Kuala Lumpur, Malaysia; 4 Centre of Public Health, Queen's University Belfast, Belfast, UK

**Keywords:** Adolescent health, cohort study, non-communicable diseases, Malaysia

## Abstract

**Background:**

The National Health & Morbidity Survey (NHMS) IV (2011) observed that the prevalence of obese children aged less than 18 years in Malaysia is 6.1% compared to 5.4% overweight and obese in NHMS III (2006). As such, this observation is of public health importance as obesity is a forewarning risk factor for chronic diseases such as type-2 diabetes, cardiovascular diseases (CVD) and certain types of cancers. This MyHeART (Malaysian Health and Adolescents longitudinal Research Team) study aims to examine risk factors of non-communicable diseases (NCD) among adolescents.

**Methods/design:**

The MyHeART study is longitudinal cohort study of 1361 schoolchildren (13-years old) attending 15 public secondary schools from the central (Kuala Lumpur and Selangor) and northern (Perak) regions of Peninsular Malaysia. The study used a stratified sampling design to select the study participants. Data collected at baseline included socio-economic, lifestyle (e.g. smoking, physical activity assessment, fitness assessment, seven-day diet history), and environmental information, anthropometric measurements, blood pressure, handgrip strength and bone mineral density. Blood samples for fasting blood glucose and lipid profiles, full blood count, renal profile, as well as bone profile and serum vitamin D were taken. This study cohort will be followed up again when participants turn 15, 17 and lastly, after a period of ten years (around the age of 27).

**Results:**

Nine percent of the adolescents from this study were obese. More male participants smoked compared to female participants (15.4% vs. 4.7%). Adolescent males had higher fasting blood glucose but the female participants had lower high density lipoprotein (HDL-cholesterol) and higher low density lipoprotein (LDL-cholesterol). In addition, adolescents from the rural area had higher fasting blood glucose, diastolic blood pressure, total cholesterol and LDL-cholesterol.

**Discussion:**

Our results demonstrated that adolescents from the rural area are at higher risk of NCDs compared to their urban counterpart. Tailor made public health interventions are highly recommended for adolescents as this may minimise the dreadful NCD burden in adulthood and health disparity between the rural and urban in the near future.

## Background

Data from the World Health Organization (WHO) shows that non-communicable diseases (NCDs) caused approximately 63% of all deaths worldwide in 2008 [1] and almost 80% of deaths in 2008 from NCDs occurred in the low- and middle-income countries. Driven by the population growth and population ageing, deaths from the NCDs are projected to increase by 15% globally between 2010 and 2020, and will account for approximately 70% of global deaths by 2030 [1-3]. It is estimated that NCDs account for 67% of all deaths in Malaysia and there has been an increasing trend in recent decades [1,2]. Non-communicable diseases are largely attributed to modifiable and non-modifiable risk factors. The Malaysian National Health and Morbidity Survey (NHMS) 2011, demonstrated that the prevalence of obesity in less than 18 years old was 6.1% (CI: 5.6-6.8), with the highest prevalence among the Malay and Indian ethnic groups. A cross sectional study has identified unhealthy dietary intake and lack of physical activity level associated with higher body mass index among the Malaysian adolescents [4]. Behavioural risk factors such as unhealthy diet, insufficient physical activity, tobacco use, and excessive alcohol consumption, contribute to the development of various metabolic diseases such as hypertension, diabetes, hypercholesterolaemia, overweight and obesity. Alarmingly, these risk factors are also becoming rampant in adolescents and consequently, contribute to a higher cost to manage and treat NCDs in the future [3,5].

Despite various campaigns and programs undertaken to reduce the prevalence of risk factors of NCDs, these risk factors continue to be a major issue in both developed and developing countries in Asia [1]. The adolescent period encompasses several transitions whereby the family, environment and societal influences are strongly associated with their health outcomes. In an attempt to address setbacks such as lack of data and poor understanding of NCDs and its risk factors in early life among the unique, multi-ethnic Malaysian population; the MyHeART study was developed as a cohort study targeting the rural and urban adolescent communities in Malaysia. The research goals include (a) identifying the prevalence and trends, of non-communicable diseases risk factors among adolescents in Peninsular Malaysia and (b) to evaluate how lifestyle factors (e.g. dietary intake and pattern, physical activities, smoking and alcohol consumption) of early adolescents influence the development of chronic NCD in their early adulthood. To the best of our knowledge, this is the first Malaysian adolescent cohort study with a representative sample size, comparable to adolescent cohort in high-income countries [7,8].

The objective of this study is to enable early detection and possible prevention of diseases by identifying predictors of NCDs. The outcome from this study will assist in the development of future pragmatic public health interventions to prevent NCDs in settings where resources are limited.

## Methods

### Study area and population

**Figure 1:**
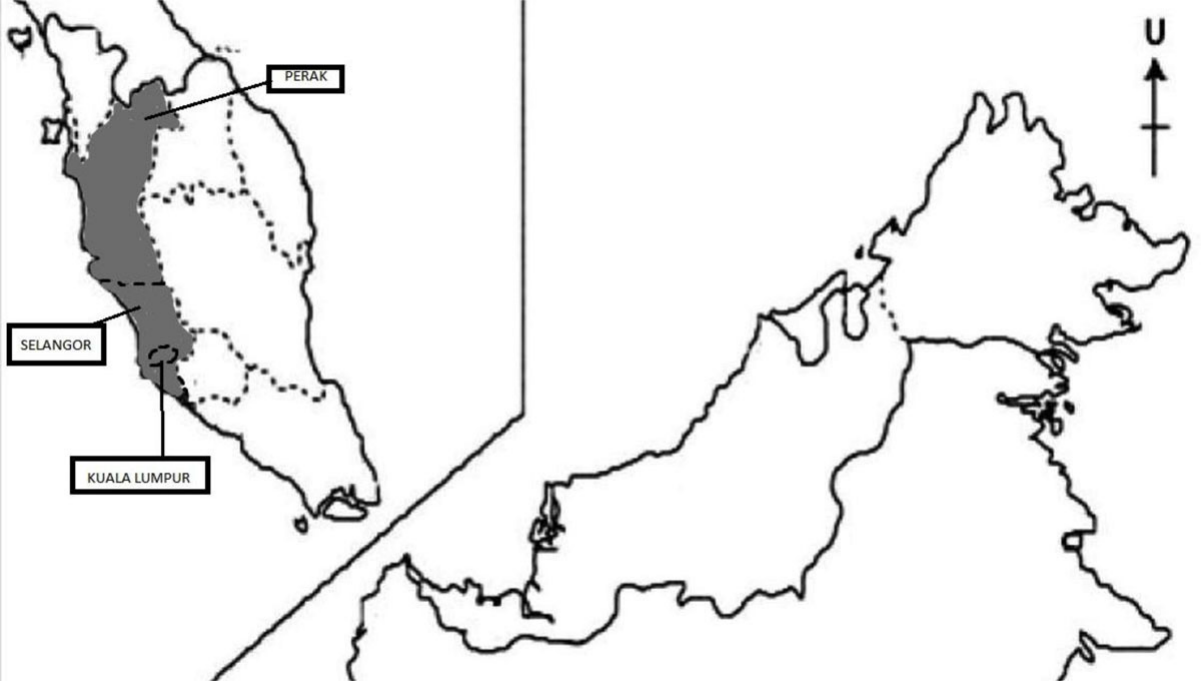
**Map of Malaysia highlighting the study area**.

This prospective cohort study was designed to recruit 1500 secondary school students from the three states in the central and northern region of Peninsular Malaysia, namely Selangor, Federal Territory of Kuala Lumpur and Perak (Figure 1). Notably, Selangor is one of the most developed and populous states in Malaysia followed by Perak. The Federal Territory of Kuala Lumpur (KL), directly governed by the federal government of Malaysia comes in third rank in regards to population although its capital city is located within this territory. Interestingly, the Federal Territory of KL is a complete urban area, whereby the central zone is largely urban with several rural areas within the northern region.

The study population were 13 years old school children from public schools, i.e. in their first year (Form 1) of secondary school. Participants were required to be able to read Malay, the national language of Malaysia. Boarding, religious and vernacular schools were excluded, as majority of its students may encompass a single ethnic group.

### Sampling

The study followed a stratified sampling design. First, a complete list of the public secondary schools, located in the selected regions were obtained from the Ministry of Education Malaysia and used as the sampling frame. The schools were stratified into urban and rural based on the criteria provided by the Department of Statistics Malaysia. A number of schools from the urban and rural locations were randomly selected using computer-generated random number lists. All form one students (13 years old) who were able to speak and write in the national language were invited to participate in this study. Next, the participants and their parents/guardians received detailed written information about the study and consent forms. Finally, participants and their parents/guardians were required to submit completed consent forms to indicate their willingness to participate.

### Sample size calculation

The study used the stratified sampling where the following formula was applied for sample size calculation; n = (z^2 ^× p × q/ r × e^2^) × design effect (z = the standard normal deviate set at 1.96 at 5% level for two-tailed test, p = estimated prevalence, q = 1 - p, r = response rate and e = precision level). The sample size was calculated using the estimated prevalence of adolescent students aged 13-15 who smoked in school; 33% [7]. The total sample estimated was about 1500 participants.

### Data collection

#### Student and parental questionnaires

Two self-administered questionnaires were used (Table 1), a Parental Questionnaire and a Student Questionnaire. These questionnaires were adapted from the Young Hearts study, an adolescent cohort study conducted in Northern Ireland [9]. The parental questionnaire gathered information on the student's birth, health and lifestyle as a child, the parents' socioeconomic status and lifestyle, as well as personal and family history of NCDs. The student questionnaire on the other hand, collected information on demographics, lifestyle, pubertal staging, sleeping practices, satisfaction with life and behavioural factors. Detailed information was collected on smoking, alcohol consumption, online and electronic usage, as well as basic information on drug intakes and gambling. Health related information including breathing/airway and skin problems was also gathered. The Tanner Staging was referred in regards to the compilation of information on pubertal development [10]. Knowledge and awareness of sexual and reproductive health issues were also assessed.

**Table 1:** Information gathered in the MyHEART study questionnaires.

Parental questionnaire	Data
Questions relating to the child's mother	Age, height, weight, current job, highest education, health problems during pregnancy/delivery, mode of delivery, age when delivered participant, number of children, smoking history including during pregnancy, consumption of alcoholic drinks, illnesses (obesity, high blood pressure, cholesterol, angina, heart attack, stroke, diabetes, asthma, bronchitis, osteoporosis, cancer), illness of close family members, income

Questions relating to the child's father	Age, height, weight, current job, highest education, smoking history, consumption of alcoholic drinks, illnesses (obesity, high blood pressure, cholesterol, angina, heart attack, stroke, diabetes, asthma, bronchitis, osteoporosis, cancer), illness of close family members, income

Questions relating to the child	Birth weight, gestational age, infant feeding history, history of abnormal large appetite, excess weight gain, medical conditions or disabilities (congenital defects, cardiovascular problems, diabetes, asthma, allergies, mental health etc.), medication and supplement use

**Student Questionnaire**	

Demographic and student's characteristics	Name, identification card number, home address, district, postcode, state, home telephone, mobile number, email, date of birth, ethnicity, religion, gender, nationality

Lifestyle	Physical activity questionnaire (PAQ-c)[15], sleep duration and quality, satisfaction with life

Health background	Eating habits, purging, dieting, breathing problems (wheezing, asthma, treatments), usage of inhalers, nose problems unrelated to flu (eg: sneezing, blocked nose), skin problems (rash, eczema)

High risk behaviour	Smoking habits, alcohol consumption, drugs intake, online and electronic usage, gambling habits

Female sexual and reproductive health	Tanner staging system, menstrual periods, knowledge on sex

Male sexual and reproductive health	Tanner staging system, knowledge on sex

In addition, these questionnaires were translated into Bahasa Melayu (the national language). Face and content validity were also evaluated in 30 subjects prior to the commencement of study. Self-administered parental questionnaires were completed by parents at home and submitted. Some missing information in the parental questionnaire was obtained with active follow-up, conducted via telephone interview, when required. The adolescents on the other hand completed their questionnaires at school (on the study day) under the supervision of the research team who ensured no required particulars were missing.

#### Dietary assessment

Generally, habitual dietary intake can be assessed using several methods such as diet histories (24-hour dietary recall or seven-days), food frequency, three days dietary records and duplicate food weighing to evaluate energy intake. However, the seven-days diet history was chosen for this study as this method has produced more valid estimates of energy intake in children and adolescents compared to other methods [11,12]. The tool was pre-tested on 40 participants from two different schools (one school each from urban and rural area respectively). For the actual study, seven qualified and trained dietitians used open-ended interviews with the students to collect information on the food and drinks that they consumed for breakfast, mid-morning snacks, lunch, afternoon tea, dinner and supper over the previous seven day period. Illustrated flip charts containing local food were used as supplementary tools to assist the study participants during the dietary evaluation and to help estimate the portion size of the foods that they consumed [13]. Nutrient intake was calculated using the Nutritionist Pro™ Diet Analysis (Axxya Systems, US) software.

#### Physical activity

Self-reported physical activity was assessed using validated physical activity questionnaire for older children (PAQ-C) based on a Malay version, which has good internal consistency and acceptable validity [14,15]. Ten items were assessed in PAQ-C to obtain physical activity level of the adolescents in the past seven days. The first item included the type and frequency of sports or/and dance the adolescents performed during the past seven days. The second to eighth items of the questionnaire assessed the activity of the adolescents during physical education (PE) classes, recess, lunch time, right after school, evenings, weekend and leisure periods. Five point Likert scale was used to answer items two to four. Item nine included the previous week physical activity frequency and item ten to report any unusual activities during the previous week. The categorisation of PAQ-C was based on a study by Crocker *et al*. [16].

#### Physical evaluation

Height was measured without socks and shoes using a calibrated vertical steadiometer (Seca Portable 217, Seca, UK), and was recorded to the nearest 0.1 cm. Weight was measured with light clothing, using a digital electronic weighing scale (Seca 813, Seca, UK) and was recorded to the nearest decimal fraction of kilogram (0.1 kg). Body mass index (BMI) was calculated as weight in kilograms divided by the square of height in meters. Body mass index z-score for age and gender was calculated using the World Health Organization (WHO) Anthro Software version 3.2.2 for the Statistical Package for the Social Sciences (SPSS) macro, based on WHO reference 2007 (WHO, Geneva, Switzerland). Body fat composition was measured using a portable body composition analyser (Tanita SC-240 MA, Body Composition Analyser, Tanita Europe B.V., The Netherlands). Both waist circumference (WC) and hip circumference (HC) were respectively measured with a non-elastic Seca measuring tape (Seca 201, Seca, UK), to the nearest millimetre.

A calibrated hand dynamometer (Jamar, Sammons Preston Rolyan, Illinois, US) was used to perform the hand grip strength test. Participants were asked to show their dominant hand. The first test was performed with dominant hand and next, with the non-dominant hand. Three sets of test were repeated alternately for both hands. The length of the hand span was also measured from the tip of the thumb to the tip of the small finger with the hand opened as wide as possible using a non-elastic Seca measuring tape (Seca 201, Seca, UK), to the nearest millimetre. Bone mineral density was measured using a portable broadband ultrasound bone densitometer (Hologic Sahara, Hologic Inc., USA).

Medically trained personnel, paediatricians, medical officers or staff nurses, measured the blood pressure and pulse rate of the participants after an interval of five minutes between each reading. The participants sat upright with his or her right upper arm positioned at the level of the heart with both feet flat on the floor. Systolic and diastolic arterial blood pressure were obtained using a stethoscope and a mercury sphygmomanometer (CK-101C, Spirit Medical Co.,Taiwan). Three readings of blood pressure were taken with two minutes interval between each reading and the mean of the three readings were obtained.

#### Exercise test

An exercise test was performed under the close supervision of a sports physician. Participants with known medical conditions, musculoskeletal injuries, or who were acutely ill were excluded. The modified Harvard Step Test protocol (30 cm step) was used as it objectively categorises the performance level of children [17]. The participants get onto and off the step box at a pace of 30 cycles per minute with a metronome set at 120 beat per minute (bpm), for a total of five minutes. A finger pulse oximeter (Baseline 12-1926 Fingertip Pulse Oximeter, Fabrication Enterprises Inc., USA) was attached to one of the student's fingers, and the pulse rate was then continuously monitored. The peak pulse rate of each student during each minute of the step box exercise was recorded. Those with a pulse rate of 200 bpm, and those who had difficulty in breathing, or were unable to finish, were directed to stop immediately.

#### Blood profile

Participants were asked to fast for at least ten hours before the study. A total of 15 ml of fasting blood was withdrawn from each participant at baseline and subsequently collected during the follow up cohort. Blood were sent to the certified International Organization for Standardization (ISO) hospital pathology lab for analysis. The samples were temporarily stored at four degrees Celsius in a cool box immediately after the blood had been withdrawn to preserve levels of markers that are sensitive to degradation due to increase in temperatures. All samples were processed in the field laboratories in the states. Samples were spinned and stored as serum and divided into several aliquots of 0.5 ml of serum. The following tests were performed at the field laboratories: full blood count (Advia 2120 flow cytometry, Siemens, Germany), fasting blood glucose (Advia Chemistry, Siemens, Germany), renal, lipid and bone profiles (Advia Chemistry, Siemens, Germany), vitamin D and parathyroid hormone (Advia Centaur XP immunoassay, Siemens, Germany). All the aliquots for future lab analysis were stored at a temperature of 80 degree Celsius freezers at the field laboratories until they were ready to be transported back to the University of Malaya (UM) bio bank.

Additional blood sampling (three ml bloods for serum and preparation of buffy coats) will be collected in future cohorts to control errors that may arise from single sampling. All other blood measurements in this cohort will be repeated in the future cohorts.

### Proposed follow-up and outcome measurement

The cohort will be followed up at two, four and 14 years post baseline data collection. Similar assessments will be performed including the questionnaires, seven-days diet recall, anthropometric measurement, blood pressure measurement, exercise test and blood sampling. In Malaysia, each citizen is provided with a national identification (ID) card that has a unique number sequence. This identification number will be used during follow-up to ensure correct matching of longitudinal data of each individual.

### Dissemination of results to the participants

Results on full blood counts, renal profile, fasting blood glucose and lipid profile were verified by a paediatrician and disseminated to the participants' parents. The participants were informed to have a clinic follow-up at the nearest government clinics or at our institution for further management or treatment if the results were beyond the clinical reference ranges and if they require urgent medical attention.

### Data management and data access

Data entry of questionnaires and blood results were entered manually. The principal researchers had prepared the template for data entry. However, to minimise errors from manual data entry, two trained researchers cross-checked data entered by each other. Regular checking and data cleaning were implemented. Any discrepancies were notified to the data manager.

To ensure standardised data management, training was provided to field researchers who are involved in the data collection. Next, all questionnaires were checked by the field researchers for its completeness. Data are treated confidentially by removing any identification (names, addresses and national identification number) and specific ID was provided for data analysis. These confidential data are only accessible to the authorised research members. All the data are stored on the University of Malaya server. Requests for any data or information for the purpose of writing a manuscript can be made to the authorised research staff via email.

## Definitions

Overweight and obesity was defined using the International Obesity Task Force criteria with extrapolation to adult BMI cut-offs of 25 kg/m^2 ^for overweight (21.91 kg/m^2 ^for males and 22.58 kg/m^2 ^for female) and 30 kg/m^2 ^for obesity (26.84 kg/m^2 ^for male and 27.76 kg/m^2 ^for female) [18]. The cut-off value used for waist circumference at 90^th ^percentile was 83.8 cm for male and 78.8 cm for female [19]. The cut-off points for at risk of metabolic syndrome includes high density lipoprotein (HDL)-cholesterol <1.03 mmol/l, triglycerides ≥1.7 mmol/l and fasting plasma glucose ≥5.6 mmol/l or known type 2 diabetes mellitus [20]. Blood pressure is considered high if the systolic blood pressure ≥130 mmHg or diastolic blood pressure ≥85 mmHg [20].

### Data analysis and statistical methods

For data analysis, the SPSS software for Windows (Version 20.0, Chicago, IL, US) was used. Qualitative variables were described as frequencies and percentages.

For baseline comparison, the chi-square tests were used to compare groups. To compare two groups for quantitative variable, the *t*-test was used if variances are equal and non- parametric Mann-Whitney-U test were used, otherwise.

For comparing more than two groups, the ANOVA procedure was used, and where necessary, the non-parametric Kruskal-Wallis test was used. Multiple linear regressions were performed separately by gender to determine the relationship between body composition measurements and selected variables.

In analysing longitudinal data, repeated measures and generalised estimation procedures in SPSS will be used. Attempt will be made to analyse the data using latent growth models in the AMOS software.

### Ethical considerations

Ethical approval was obtained from the Medical Ethics Committee, University Malaya Medical Centre (MEC Ref. No: 896.34). The National Medical Research Register number is 14-376-20486. Participation in the study was voluntary and written informed consent and ascent for participation in the study was obtained from the parents or guardian as well as the participants.

## Results

In total, at the time of sampling, there were 238 secondary schools in the northern zone, 261 in the central zone and 96 in KL. Based on a feasibility study performed in 2011, and an estimate of recruitment of approximately 80-120 students per school, 15 schools were randomly selected for the study, five in Selangor, seven in Perak and three in KL; eight were urban schools and seven rural. There were 2694 eligible participants within the schools and 1361 voluntarily participated between March and May 2012, giving an overall response rate of 51%. Urban schools response rate ranges from 22%-53% whilst rural schools response rates range from 40%-84%.

The distribution of the participants in terms of gender, ethnicity and location (urban and rural) and key risk factors for chronic non-communicable diseases including hypertension, fasting blood glucose, hypercholesterolaemia, overweight/obesity and smoking are shown in Table 2.

**Table 2:** Demographic Characteristics and prevalence of parameters for adolescent aged 13 years old by gender and Place of Residences

Baseline characteristic	Male	Female	Total (%)	Chi square	
	
(N = 1361)	N = 525 No (%)	N = 836 No (%)		x²	p-value
**Place of residence**					
Urban	240 (45.7)	483 (57.8)	723 (53.1)	18.8	<0.001
Rural	285 (54.3)	353 (42.2)	638 (46.9)		
**Systolic Blood Pressure (mmHg)**					
Normal (<130)	497 (95.2)	795 (95.1)	1292 (94.9)		
Hypertensive (≥130)	25 (4.8)	33 (3.9)	58 (4.3)	0.5	0.478
**Diastolic Blood Pressure (mmHg)**					
Normal (<85)	496(94.5)	788 (94.3)	1284 (94.3)		
Hypertensive (≥85)	26 (5.0)	40 (4.8)	66 (4.8)	0.02	0.901
**Fasting Blood Glucose (mmol/L)**					
Normal (3.9-5.5)	496 (94.5)	788 (94.3)	1284 (94.1)	9.3	<0.05
High (≥5.6)	26 (5.0)	27 (3.2)	53 (3.9)		
Low (<3.9)	3 (0.6)	21 (2.5)	24 (1.8)		
**Total Cholesterol (mmol/L)**					
Desirable (<5.2)	415 (79.0)	624 (74.6)	1039 (76.3)	3.5	0.176
Borderline (5.2-6.2)	92 (17.5)	178 (21.3)	270 (19.8)		
High Risk (>6.2)	18 (3.4)	34 (4.1)	52 (3.8)		
**HDL Cholesterol (mmol/L)**					
At risk (≤1.03)	478 (91.0)	795 (95.1)	1273 (93.5)	8.74	<0.05
Normal (>1.03)	47 (9.0)	41 (4.9)	88 (6.5)		
**LDL Cholesterol (mmol/L)**					
Optimal (<2.58)	252 (48.0)	334 (40.0)	586 (43.1)		
Near Optimal (2.58-3.34)	179 (34.1)	343 (41.0)	522 (38.4)		
Borderline (3.35-4.11)	73 (13.9)	120 (14.4)	193 (14.2)	9.74	<0.05
High (4.12-4.89)	15 (2.9)	31 (3.7)	46 (3.4)		
Very high (>4.9)	6 (1.1)	8 (1.0)	14 (1.0)		
**Triglycerides (mmol/L)**					
Normal (<1.7)	491 (93.5)	784 (93.8)	1275 (93.7)	0.04	0.850
High (≥ 1.7)	34 (6.5)	52 (6.2)	86 (6.3)		
**BMI (IOTF standards kgm^-2^)**					
Underweight (<15.8^m^; <16.3^f ^)	103 (20.0)	183 (23.0)	286 (21.9)		
Normal (15.8 - <21.9^m^; 16.3 - <22.6^f^)	282 (53.7)	456 (54.5)	738 (54.2)	5.8	0.329
Overweight (<21.9 - <26.8^m^; 22.6 - <27.8^f^)	82 (15.6)	128 (15.3)	210 (15.4)		
Obese (≥26.8^m^; ≥27.8^f^)	56(10.7)	60 (7.2)	116 (8.5)		
**Smoking**					
No	432 (82.3)	790 (94.5)	1222 (89.8)	47.8	<0.001
Yes	81 (15.4)	39 (4.7)	120 (8.8)		

	**Urban**	**Rural**	**Total (%)**	**Chi square**	
	**No (%)**	**No (%)**		**x^2^**	**p-value**

**Ethnicity**					
Malay	521 (72.1)	570 (89.3)	1091 (80.2)		
Chinese	98 (13.6)	7 (1.1)	105 (7.7)	118.5	<0.001
Indian	83 (11.5)	22 (3.4)	105 (7.7)		
Others	12 (1.7)	29 (4.5)	41 (3.0)		
**Systolic Blood Pressure (mmHg)**					
Normal (<130)	680 (94.1)	612 (95.9)	1292 (94.9)		
Hypertensive (≥130)	32 (4.4)	26 (4.1)	58 (4.3)	0.14	0.705
**Diastolic Blood Pressure (mmHg)**					
Normal (<85)	687 (95.0)	597 (93.6)	1284 (94.3)		
Hypertensive (≥85)	25 (3.5)	41 (6.4)	66 (4.8)	6.1	<0.05
**Fasting Blood Glucose (mmol/L)**					
Normal (3.9-5.5)	692 (95.7)	592 (92.8)	1284 (94.1)	10.7	<0.05
High **(≥**5.**6**)	26 (3.6)	27 (4.2)	53 (3.9)		
Low (<3.9)	5 (0.7)	19 (3.0)	24 (1.8)		
**Total Cholesterol (mmol/L)**					
Desirable (<5.2)	579 (80.1)	460 (72.1)	1039 (76.3)		
Borderline (5.2-6.2)	127 (17.6)	143 (22.4)	270 (19.8)	15.6	<0.001
High Risk (>6.2)	17 (2.4)	35 (5.5)	52 (3.8)		
**HDL Cholesterol (mmol/L)**					
At risk (≤1.03)	681 (94.2)	592 (92.8)	1273 (93.5)	1.1	0.294
Normal (>1.03)	42 (5.8)	46 (7.2)	88 (6.5)		
**LDL Cholesterol (mmol/L)**					
Optimal (<2.58)	343 (47.4)	243 (38.1)	586 (43.1)		
Near Optimal (2.58-3.34)	271 (37.5)	251 (39.3)	522 (38.4)	27.9	<0.001
Borderline (3.35-4.11)	93 (12.9)	100 (15.7)	193 (14.2)		
High (4.12-4.89)	10 (1.4)	36 (5.6)	46 (3.4)		
Very high (>4.9)	6 (0.8)	8 (1.3)	14 (1.0)		
**Triglycerides (mmol/l)**					
Normal (<1.7)	670 (92.7)	605 (94.8)	1275 (93.7)	2.67	0.102
High (≥ 1.7)	53 (7.3)	33 (5.2)	86 (6.3)		
**Smoking**					
No	656 (90.7)	566 (88.7)	1222 (89.8)	1.26	0.262
Yes	58 (8.0)	62 (9.7)	120 (8.8)		

m=male, f=female

Table 2 shows the breakdown of the participants in terms of age, sex, ethnicity, location (urban or rural) and the prevalence of cardiovascular risk factors. There were more female participants than male participants and higher number of participant were from the urban schools compared to rural. There were more Malay compared to the other two ethnic groups as Malay is the major ethnic group in Malaysia. The overall prevalence of reported smoking was 8.8%, out of which 15.4% represented male participants and 4.7% represented females participants. The overall overweight/obese prevalence among the adolescents in this study was 23.9%, with 8.5% of them obese and 15.4% overweight.

## Discussion

The baseline data collected in the MyHeART study showed the prevalence of overweight in Malaysian adolescents was around 15% for both males and females with the prevalence of obesity in males recorded higher percentage than the females (10.7% vs. 7.2%). Furthermore, our studies have shown an increasing trend of overweight children aged 1-12 years from 20.7% in 2002 to 26.5% in 2008 within the Peninsular Malaysia (National Strategic Plan 2010) and a higher prevalence of 34.2% was reported for the prevalence of overweight and obese children in metropolitan Kuala Lumpur [21]. The prevalence of overweight and obese Malaysian adolescents is also higher than other South East Asian countries such as the Philippines' (4.8%) [22] and Thailand (16.6%) [23]. Hence, this is certainly alarming as the trend of overweight and obesity for developing country like Malaysia is mirroring countries like England i.e., following an increasing trend where 11% of male and 12% of female aged 2 until 15 years were obese [24]. In addition, the prevalence of overweight or obese adolescents in the United States for those aged 12 to 19 years was also high, 35% compared to 4.6 % in 1963 [25].

Evidences of childhood obesity leading to complications related to obesity in adulthood [25,26] remain consistent and urbanisation may contribute to the rise of overweight and obese population in Malaysia. Although the worldwide study (n = 19244) revealed that adult obesity in developed countries have slowed down, the prevalence of obesity among adults in developing countries is still disturbing, exceeding 50% [27]. The influence of urbanisation as a result of high economic growth has undoubtedly contributed to the prevalence of overweight and obese adolescents in China [28], India [29], Argentina [30] and Poland [31]. Similarly, the rapid economic growth and urbanisation may have played a crucial role in the increased prevalence of overweight and obesity in Malaysia.

The prevalence of cardiovascular risk factors (high fasting blood glucose and smoking) was found to be higher in male participants compared to female participants. However, female participants were noted to have lower HDL-cholesterol (a protective cardiovascular risk factor). Adolescents from the rural areas were found to have higher fasting blood glucose, diastolic blood pressure, total cholesterol and low-density lipoprotein (LDL)-cholesterol levels compared to their urban counterpart. Hence, it is vital to monitor and screen these adolescents especially those in rural areas since these markers of metabolic syndrome are strongly associated with early cardiac disease and type-2 diabetes mellitus [32-34].

On a positive note, there are several strengths that can be highlighted from this study. To our knowledge, this is the first adolescent cohort study to be conducted in Malaysia that has a comprehensive anthropometric assessment, complete fasting blood profile and dietary assessment. At present, there is one large adult cohort similar to this study that was conducted in Malaysia i.e. The Malaysian Cohort [35]. As such, our study may contribute to the lack of knowledge in regards to the health status among the younger generation of Malaysians. Moreover, most studies conducted on school children in Malaysia were cross-sectional studies, as such previous analyses may not be able to capture the progression and variation of biomarkers, anthropometric measurements, nutritional status and diet among the adolescent cohort. Next, this study used stratified sampling to ensure adequate recruitment of participants from both urban and rural. As such, our study enabled us to investigate health disparity between rural and urban adolescents. Although seven-days diet history is the most reliable tool to assess energy intake for this group, its implementation requires a lot of resources and it is costly. By having a validated food frequency questionnaire (FFQ), resources can be minimised and simultaneously enable the dietary intake to be reported by the respondents independently. To date, there is only one validated FFQ for adolescents in Malaysia with subjects recruited exclusively from one state and were predominantly Malays [36]. The data that we gathered from this study using the seven-days diet history on the other hand will assist us in the development of a more robust FFQ for adolescents. This new FFQ will be validated against the biomarkers.

On the contrary, despite all the advantages mentioned above, the first cohort has several limitations such as low representative of the Chinese and Indian ethnic groups. Thus for the next cohort, it is necessary to oversample the Chinese and Indian ethnic groups for further recruitment. Next, questionnaires were entered manually and therefore, for future cohorts, it would be beneficial to use software [37] that can classify paper forms and convert the required data into usable digital information, enabling researchers to extract information easily.

Results from this cohort may potentially help the stakeholders and researchers to conduct and evaluate appropriate intervention that may give an impact towards the improvement of adolescents' health status and consequently for their adulthood. This will also lead to the development of health policies that will influence the national strategic plan for better health outcome thus minimising the treatment cost of the chronic NCDs. To ensure this cohort study is a successful venture, careful considerations and appropriate strategies should be implemented. Few important considerations to minimise the time that may affect participants study time were by conducting the recruitment systematically during physical education day and recruiting participants between February and June to ensure the recruitment period is far from the examination months. The school team involvement in planning and implementing future cohorts is important, and this is constantly being built up by building good rapport between researchers and the school team.

In conclusion, it appears that adolescents' male participants in this cohort have higher fasting blood glucose and reported smoking compared to female participants at a young age of 13 years old, with the females having lower HDL-cholesterol. This study also revealed that the adolescents from the rural area had more non-communicable diseases risk factors. The result of this study is hoped to gain the attention of the public health sector to develop more effective measures in schools to minimise this present health gap.

## Competing interests

The authors declare that they have no competing interests.

## Authors' contributions

All authors contribute to the study design; MAH, MYJ, NAS and TTS were involved in the field work and data collection. MAH was responsible for the drafting of this manuscript and all authors approved the final manuscript.
